# MicroRNA analysis suggests an additional level of feedback regulation in the NF-κB signaling cascade

**DOI:** 10.18632/oncotarget.4005

**Published:** 2015-05-16

**Authors:** Peter Mechtler, Ruchi Singhal, Julia V. Kichina, Jonathan E. Bard, Michael J. Buck, Eugene S. Kandel

**Affiliations:** ^1^ Department of Cell Stress Biology, Roswell Park Cancer Institute, Buffalo, NY, USA; ^2^ Department of Biochemistry, State University of New York, Center of Excellence in Bioinformatics and Life Sciences, Buffalo, NY, USA; ^3^ Cellecta, Inc., Mountain View, CA, USA

**Keywords:** RIPK1, miR-497, miR-146a, miR-215, IKKβ

## Abstract

It is increasingly clear that the biological functions of a transcription factor cannot be fully understood solely on the basis of protein-coding genes that fall under its control. Many transcription factors regulate expression of miRNAs, which affect the cell by modulating translation and stability of mRNAs. The identities and the roles of NF-κB-regulated miRNAs have been attracting research interest for a long time. We revisited this issue in a system with controlled expression of one of the key regulators of NF-κB, RIPK1. Several regulated miRNAs were identified, including miR-146a, miR-215 and miR-497. The miRNAs were also inducible by IL-1β, but not when NF-κB activity was repressed by mutant IκBα. The presence of a miR-497 site was predicted in the 3′-UTR of IKBKB gene, which encodes IKKβ. Using appropriately engineered reporters, we confirmed that this site can be a target of suppressive action of miR-497. Our findings suggest that NF-κB controls expression of a miRNA, which may reduce production of IKKβ. Considering the role of IKKβ in the canonical pathway of NF-κB activation, our observations may indicate a new mechanism that modulates the magnitude of such activation, as well as the propensity of a cell to engage canonical vs. non-canonical pathways.

## INTRODUCTION

MiRNAs are short non-coding RNAs predominantly known for attenuating translation of various protein-coding RNAs [1]. Changes in the repertoire of miRNAs accompany such diverse phenomena as aging [2, 3], cognitive impairment [4], and oncogenesis [5, 6], and it is widely accepted that miRNAs control major physiological and pathophysiological processes. MiRNAs are generated from longer transcripts via a series of nucleolytic processing steps [7]. Each mature miRNA may target multiple mRNAs [8]. Short stretches of homology between the miRNA and the mRNA are believed to mediate this interaction, but the precise criteria that determine the specificity of this interaction are still unknown [9]. The repertoire and the abundance of miRNAs in a given cell are tightly regulated. In part, this is effected through RNA-processing enzymes [10], but the best studied mode of such a regulation is at the level of transcription [1]. A large fraction of all miRNAs are generated from RNA polymerase II-driven transcripts [11], and the same transcription factors that control protein-coding genes affect miRNA expressions as well. Indeed, it could be argued that a biological role of a transcription factor cannot be fully understood without considering the spectrum of non-protein-coding RNAs that it controls.

The signaling pathway, which culminates in activation of NF-κB family of transcription factors, was among the first to be explored for the effects on miRNA expression [12]. Typically, NF-κB factors act as dimers, which could be formed by various members of the family. RelA, RelB and c-Rel subunits possess both transactivation and DNA-binding activity, while p50 and p52 subunits can bind DNA, but are incapable of transactivation on their own. The activation of NF-κB is a key feature of immune response and inflammation [13]. The respective regulatory pathway is stimulated by a number of cytokines and other secreted factors, by pathogens and xenobiotics, as well as by certain intracellular stressors. When activated, NF-κB promotes expression of genes that contribute to immune response, angiogenesis, cell survival and many other processes [13]. Deregulation of NF-κB has been reported in autoimmune and inflammatory disorders [14–16] and aging [17, 18]. Elevated activity of NF-κB is also often observed in human malignancies, where it is believed to contribute to growth, survival and spread of the cancer cells [19].

NF-κB is under an intricate control, which still remains a subject of intense research. The centerpiece of the canonical pathway of NF-κB activation is a protein kinase called IKK [20]. This enzyme, which is composed of IKKα, IKKβ and IKKγ subunits, phosphorylates IκB (“inhibitor of kappa B”) proteins. The latter normally act to sequester NF-κB in the cytoplasm. Upon phosphorylation, IκB is targeted for degradation, while released NF-κB translocates towards its target genes in the nucleus.

A so-called “non-canonical” pathway relies on IKKα, but not the full IKK, to enhance production of NF-κB subunit p52 from its inactive precursor [21]. This eventually results in accumulation of p52/RelB dimer in the nucleus. Inducers of the canonical pathway are plentiful, with tumor necrosis factor alpha (TNFα) and interleukin-1 beta (IL-1β) being among the most notable examples [22]. Activation of the non-canonical pathway has been attributed to such molecules as lymphotoxin-α and BAFF [21]. Since various NF-κB dimers have overlapping, but not identical sets of targets, the distinction between canonical and non-canonical pathways may explain some of the differences in the biological functions of NF-κB-activating stimuli. Importantly, among the transcriptional targets of NF-κB are both the negative and the positive regulators of this pathway [23], and evidence of complex cross-talk between the canonical and non-canonical pathways have been reported [23, 24].

While the interest towards identifying NF-κB target genes and their roles has remained steady over many years, the tools available to explore these issues have been rapidly evolving. For example, an early survey of NF-κB-inducible miRNAs was limited to an array of 200 human miRNAs [12]. In contrast, miRBase, an online database of known miRNAs [25], currently contains approximately 2000 unique entries corresponding to Homo sapiens. In many cases, the ranks of the previously known miRNAs were expanded due to the discovery of new molecules. In other cases, what used to be considered a single miRNA species, proved to be a family of closely-related sequences, which originate from distinct genes, but are hard to distinguish in conventional array hybridization assays. Modern high-throughput sequencing techniques, coupled with powerful instruments of sequence analysis, are primarily responsible for the increased breadth and sensitivity of detection of small RNAs. The availability of these tools facilitates continuous exploration of miRNA profiles in new experimental systems, but also warrants a fresh look at the effects of previously identified regulators of miRNA expression.

RIPK1 protein is a central and essential element of multiple signaling pathways, including those initiated by TNFα, various xenobiotics and stress factors [26]. Depending on the context, RIPK1-dependent signaling leads to inflammation, necroptosis or apoptosis [26]. TNFα, acting through its cognate receptor and RIPK1, is among the best-studied activators of NF-κB [27]. In earlier attempts to identify events that may lead to NF-κB deregulation, we observed that overexpression of both full-length and short forms of RIPK1 mimic many aspects of TNFα treatment and sustains constitutive activation of NF-κB [28, 29]. In the current study, we identify miRNAs that are up-regulated following up-regulation of short RIPK1, and we document that these miRNAs are induced by other NF-κB-stimulating events in an NF-κB-dependent manner. Furthermore, we demonstrate that one of the induced miRNAs, miR-497, is a likely negative regulator of IKKβ, providing yet another layer of regulation for the NF-κB signaling cascade.

## RESULTS

### Identification of RIPK1-inducible miRNA

We used sequencing-based high-throughput small RNA profiling technique to identify RIPK1-dependent changes in miRNA abundance. We relied on a previously established cell line (“clone 3B4.1”), which contains a tetracycline-regulated promoter inserted into RIPK1 gene [28]. Clone 3B4.1 was originally selected for high NF-κB activity from among C6TA4 cells, which were mutagenized with a promoter-bearing transposon [28]. In turn, C6TA4 are derived from HEK293 cells, pre-engineered with a tetracycline transactivation protein and two NF-κB-responsive markers (Zeocin resistance and ganciclovir sensitivity) [30, 31]. The inserted promoter in 3B4.1 clone drives expression of a shorter form of RIPK1 protein, which potently activates NF-κB and mimics other effects of TNFα treatment [28]. Importantly, exposure to tetracycline or doxycycline inhibits the promoter and, eventually, brings the activity of NF-κB down to a basal level for the parental cell line [28].

**Figure 1:**
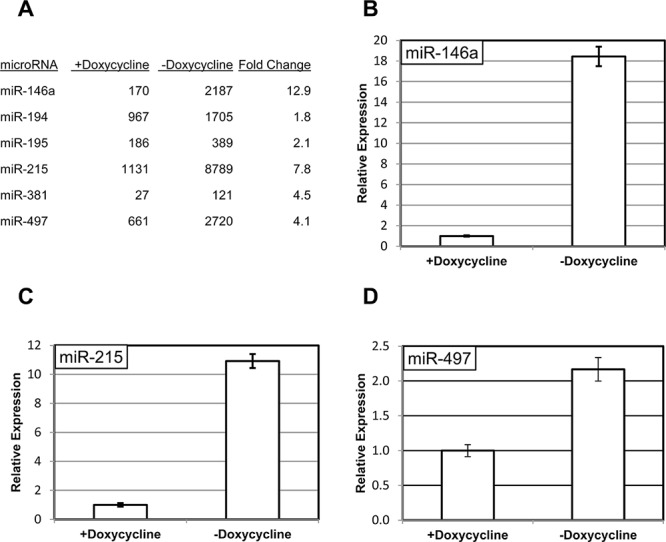
RIPK1-dependent changes in miRNA expression **A.** Examples of miRNA affected by the short form of RIPK1 cells in 3B4.1 cells. The protein is expressed under a strong promoter, which is suppressed by addition of doxycycline. The miRNAs were quantified by sequencing, as described in Materials and Methods. **B–D.** Confirmation of changes in the expression of miR-146a (B), miR-215 (C), miR-497 (D) using quantitative PCR. The levels of the miRNAs from triplicate experiments were normalized to those of an internal control (RNU6B) in the same samples and are shown relative to those in the doxycycline-treated cells.

The RNA samples from 3B4.1 cells with and without doxycycline treatment were enriched for small RNAs using size-fractionation and profiled using high-throughput sequencing as described in Materials and Methods (Supplementary Table 1). We have attempted to confirm the observed differences in the expression of miR-14a, -215, -381 and -497 (Figure 1A) using quantitative PCR. In accordance with the sequencing results, the expression levels of three of the miRNAs (miR-146a, miR-215 and miR-497) were sensitive to manipulation of RIPK1 expression (Figure 1B, Figure 1C and Figure 1D), and the differences between doxycycline-treated and untreated samples were significant for each of these miRNAs (*p* < 0.05). Importantly, miR-146a is a well-known transcriptional target of NF-κB [12], and may serve as an internal control for activation of the corresponding signaling cascade in our experimental conditions. The fourth miRNA, miR-381, was undetectable in these conditions (data not shown). Considering the low number of corresponding reads in the sequencing experiment, we find it very likely that miR-381 is expressed at a very low level and is unlikely to play a significant biological role in our experimental system.

### The role of NF-κB in the regulation of miR-146a, miR-215 and miR-497

While NF-κB is prominently induced by RIPK1 in 3B4.1 cells, it is not the only signaling cascade activated in these cells. We undertook two complementary approaches in order to verify that the induction of the identified miRNAs is, indeed, NF-κB-dependent.

**Figure 2:**
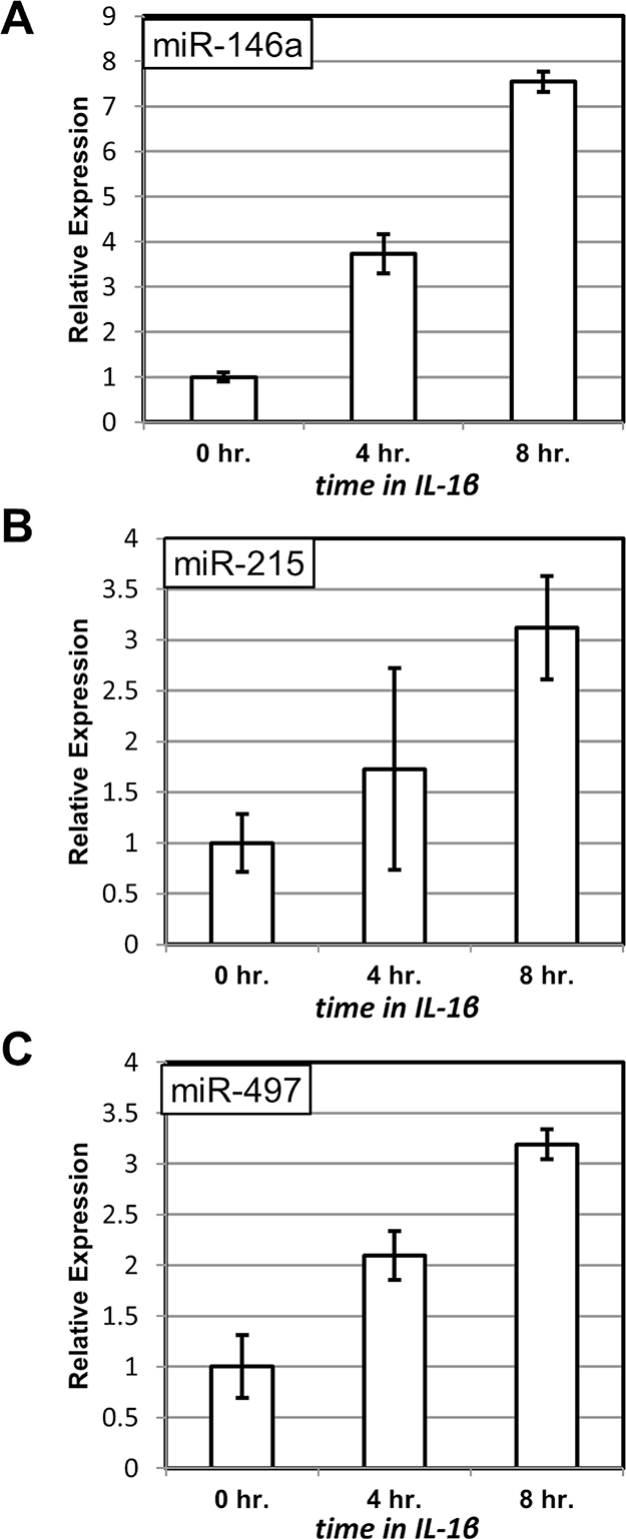
Induction of miRNA expression upon treatment with IL-1β The levels of miR-146a **A.** miR-215 **B.** and miR-497 **C.** were measured by quantitative PCR at the indicated times after addition of IL-1β to C6TA4 cells. The levels of the miRNAs from triplicate experiments were normalized to those of an internal control (RNU6B) in the same samples and are shown relative to those in untreated cells.

First, we examined whether the same miRNAs are inducible by IL-1β. TNFα, whose action is mimicked by overexpression of RIPK1 in 3B4.1 cells, and IL-1β signal through distinct intermediates and elicit non-identical responses in the affected cells. However, both cytokines are powerful inducers of NF-κB activity. Accordingly, we observed that IL-1β treatment of C6TA4 cells resulted in elevated expression of miR-146a, miR-215 and miR-497 (Figure 2A, Figure 2B and Figure 2C, respectively). The differences between samples at the start (0 hr.) and at the end (8 hr.) of the experiment were significant (*p* < 0.05) for all the three miRNAs.

**Figure 3:**
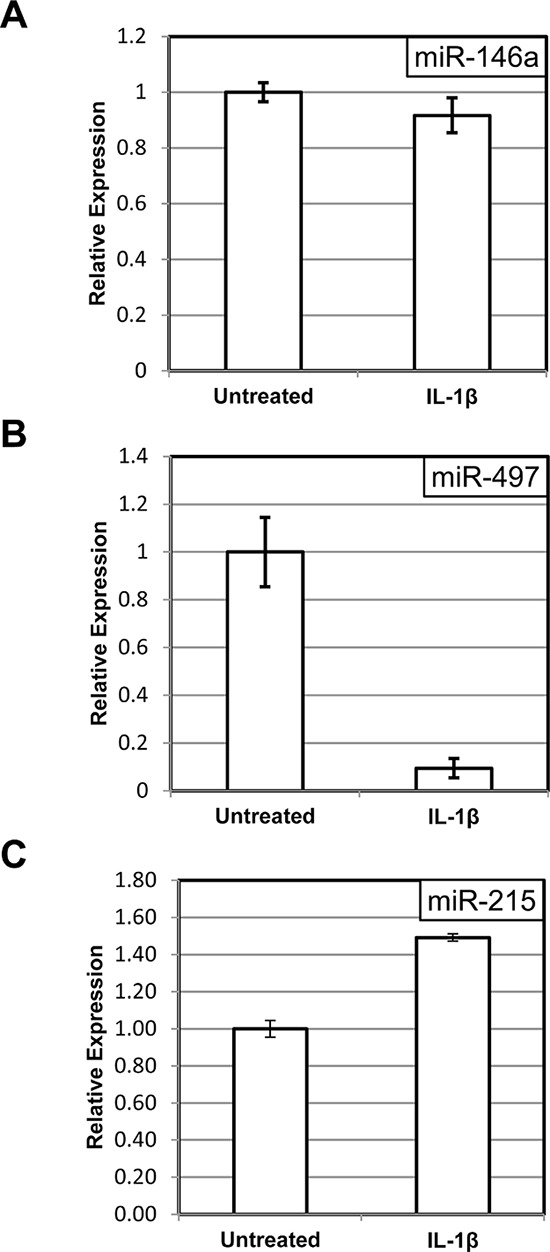
“Super-repressor” mutant of IκBα prevents miRNA induction by IL-1β The levels of miR-146a **A.** miR-497 **B.** and miR-215 **C.** were measured in C6TA4 cells stably transduced with a stabilized (“super-repressor”) mutant of IκBα, with or without an eight-hour treatment with IL-1β. The experiments were performed and analyzed as in Figure 2.

Second, we examined how the induction of miR-146a and miR-497 is affected by the presence of a “super-repressor” mutant of IκBα, which lacks the phosphorylation site of IKK. The unphosphorylatable mutant cannot be targeted by upstream signals for degradation and, hence, continues to sequester NF-κB in the cytoplasm even upon stimulation with cytokines [32]. Accordingly, we have previously observed that C6TA4 cells harboring mutant IκBα fail to activate NF-κB and are hypersensitive to pro-apoptotic effects of TNFα [28]. As expected, the ability of IL-1β to induce the miRNAs was diminished when the mutant IκBα was expressed (compare Figure 2A to Figure 3A, Figure 2B to Figure 3B, and Figure 2C to Figure 3C), and this effect was significant (*p* < 0.05) for all the three miRNAs.

In fact, when NF-κB activity was suppressed, miR-497 expression was reduced, rather than elevated, following IL-1β treatment (Figure 3B). A small increase in miR-215 levels after IL-1β treatment in these cells (Figure 3C) may reflect a contribution of an NF-κB-independent mechanism or a residual NF-κB activity, which remains despite expression of the IκBα mutant.

Overall, our observations suggest that miR-146a, miR-215 and miR-497 are inducible by various stimuli in an NF-κB-dependent manner.

### MiR-497 targets a site in the 3′-UTR of IKKβ mRNA

**Figure 4:**
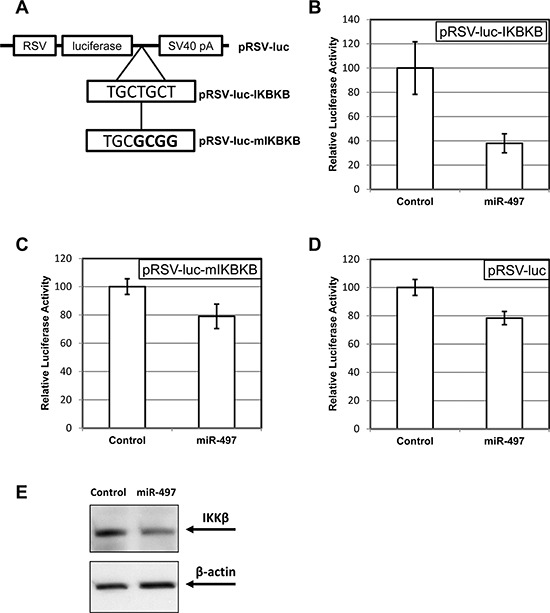
MiR-497 exhibits an inhibitory activity through the cognate site in the 3′-UTR of IKBKB (IKKβ) gene **A.** A schematic depiction of the basic luciferase reporter (pRSV-luc), the same reporter supplied with 1.3kb fragment from IKBKB 3′-UTR carrying the predicted miR-497 recognition site (pRSV-luc-IKBKB), and the construct, in which the site has been mutated (pRSV-luc-mIKBKB; mutated bases shown in bold). **B–D.** The effect of miR-497 co-transfection on the luciferase expression from pRSV-luc-IKBKB (B), pRSV-luc-mIKBKB (C) and pRSV-luc (D) The activity of luciferase in transiently transfected C6TA4 cells was normalized to that of a co-transfected constitutive β-galactosidase reporter and compared between the cells transfected with miR-497 or a non-targeting control as described in Materials and Methods. **E.** The effect of miR-497 transfection on IKKβ level in C6TA4 cells. The cells were transfected with miR-497 or a negative RNAi control. Lysates were prepared 48 h post-transfection and probed by immunoblotting for the indicated proteins. Images from one of three consistent blots are shown.

We used TargetScan [33] to predict possible targets of miR-497. The analysis revealed the presence of a putative miR-497 binding site in the 3′-UTR of IKKβ mRNA (encoded by IKBKB gene). We have tested this prediction by fusing the 3′-UTR fragment of IKKβ mRNA to the coding region of firefly luciferase (Figure 4A). The resulting construct demonstrated a profound sensitivity to the presence of miR-497 in transient transfection assays (Figure 4B), a feature not seen with the original luciferase vector (Figure 4D). The latter observation indicates that the phenomenon was not due to a generic effect on the function of the RSV promoter or the luciferase itself. Furthermore, the inhibitory effect of miR-497 was greatly reduced when the putative miR-497 site in the IKKβ UTR was mutated (Figure 4C). The fraction of pRSV-luc expression remaining after miR-497 co-transfection was significantly (*p* < 0.05) different from that of pRSV-luc-IKBKB, but not of pRSV-luc-mIKBKB. We concluded that the miR-497 site in the 3′-UTR of IKKβ mRNA acts as a bona fide regulator of protein expression in response to elevated levels of this miRNA.

Accordingly, transfection of miR-497 into C6TA4 cells resulted in a noticeable reduction of the levels of IKKβ protein, as seen by Western blotting (Figure 4E).

## DISCUSSION

HEK293-derived cells, akin to the ones used in the current study, have been extensively exploited to study the function and regulation of NF-κB (e.g. [28–31, 34]). In particular, there is evidence that overexpression of the short RIPK1 variant in clone 3B4.1 mimics many NF-κB-dependent and -independent consequences of TNFα treatment [28], and a transcript, which closely resembles the one being overexpressed in these cells, may naturally originate from intron 6 of RIPK1 gene [28]. It is important to note that the exact pattern of NF-κB-responsive miRNA genes is likely to vary depending on the biochemical, genetic and epigenetic environment of a cell, such as the abundance of various regulatory proteins (including individual NF-κB subunits), mutations and polymorphisms within promoter elements, as well as processing of the latter by chromatin-modifying enzymes. Accordingly, not all miRNAs, previously reported as NF-κB-responsive [35], were affected in our experiment (Supplementary Table 1). Additional factors contributing to such an apparent discrepancy could be a superimposition of multiple regulatory mechanisms, an inability to distinguish some closely-related miRNAs, which are encoded by distinctly regulated genes, and sampling errors due to very low expression of some of these molecules.

In the current study we specifically confirmed NF-κB-dependent regulation of three human miRNAs: miR-146a, miR-215 and miR-497. MiR-146a is a well-known transcriptional target of NF-κB [12]. This miRNA has attracted considerable attention due to its possible involvement in immunity, hematopoiesis and cancer [36]. The presence of a known NF-κB target on our list may serve as an internal control for activation of the corresponding signaling cascade in our experimental conditions. To the best of our knowledge, our study is the first to describe miR-215 and miR-497 as NF-κB targets. Importantly, miR-194 and -195, which are clustered together with miR-215 and miR-497 respectively, are also up-regulated in our experiments (Figure 1). This coordinated enrichment of clustered miRNAs in our experiments further supports the notion of bona fide transcriptional regulation of the corresponding genes. However, the precise molecular mechanisms of this regulation remain to be elucidated. The promoters of the corresponding miRNA genes have not been exhaustively characterized, and there is a formal possibility that the effect of NF-κB on their expression is indirect. Very intriguing is the observation that in the absence of NF-κB, IL-1β reduces the abundance of miR-497 (Figure 3B). We can only speculate about the mechanism of this phenomenon. For example, it is possible that in IL-1β-stimulated cells NF-κB competes with a concomitantly activated transcriptional inhibitor; or that NF-κB provides a transcriptional activation domain to a DNA-bound complex, which otherwise interferes with basal transcription. It is also important to note that the abundance of a miRNA could be controlled post-transcriptionally, at the level of processing or stability.

It is commonly accepted that control of miRNA expression is an indelible function of many, if not all, transcription factors. Studies of the relationship between transcription factors and miRNAs reveal a recurrent theme of mutual regulation. MiRNAs commonly target the signaling pathway, or even the very transcription factor, which is primarily responsible for their own expression [37]. Such a mechanism has been validated or, at least, proposed for numerous transcription factors, such as SRF [38], E2F [39], FOXO [40] and others. Indeed, miR-146a represents a classic example of such a negative feedback loop [24]: its targets, TRAF6 and IRAK1, which are key elements of several NF-κB-activating pathways.

MiR-215 is often characterized as a putative tumor suppressor, decreased in cancer and capable of inhibiting cell proliferation [41–45], although the nature of the most relevant targets in this case remains open for debate. However, this anti-cancer role does not appear to be universal, as there are reports of malignancies with miR-215 overexpression in cells, as well as of accelerated cell growth upon miR-215 overexpression [46, 47]. MiR-215 (but not the co-clustered miR-194) is reportedly upregulated by differentiation-inducing protein CDX1 and targets BMI to inhibit stemness in enterocytes [48]. The possibility that members of the same cluster could be regulated either coordinately or independently may account for some of the controversy about the correlation between miR-215 expression and cancer-related phenotypes in various systems. Of note, a sequence complementary to miR-215 seed (UCCAGU) is also present in RIPK1 mRNA. The functional significance of this observation is yet to be validated, but it opens up an intriguing possibility that elevated miR-215 expression accounts for an apparent discordance between RIPK1 mRNA and protein levels in 3B4.1 clone upon induction of the short form of RIPK1 [28].

Targeting of IKKβ by miR-497 may be viewed as yet another example of a negative feedback loop involving a miRNA and a pathway that regulates its expression. Indeed, it might be one of the contributors to the oscillatory waves of NF-κB activity, which are observed in some systems upon cytokine stimulation [49]. However, the consequences of specific reduction in the abundance of IKKβ are likely to be more complex. While the protein is essential for canonical NF-κB signaling, it is dispensable for the non-canonical pathway. Consequently, we would like to speculate that elevated expression of miR-497 would result not in a mere reduction of NF-κB activity, but in an altered propensity of a cell to activate one or the other branch of the NF-κB pathway. If the level of IKKα remains stable, while IKKβ declines, one may expect the cell to possess a larger pool of free IKKα and, hence, mount a stronger response toward non-canonical NF-κB stimuli and a weaker one towards the canonical ones. Hence, the type and the magnitude of NF-κB response may depend on the prior exposure of a cell to miR-497-inducing factors. Furthermore, a switch towards predominantly non-canonical response might ensue from re-challenging or prolonger treatment with the same cytokine. It would be interesting to explore whether the systems where a positive feedback loop is formed via NF-κB-dependent production of NF-κB-activating cytokines [30] also change the balance between the canonical and non-canonical pathways over time. Intriguingly, a gradual switch from canonical to non-canonical pathway has been well documented upon prolonged treatment with lymphotoxin-β [24]. The ability to reduce IKKβ levels may give miR-497 a beneficial role during aging, since selective loss of IKKβ is associated with reduced inflammation and a better health status in aging animals [50], while the decline in miR-497 expression is seen in a mouse model of accelerated aging [51]. Of course, ultimately the physiological role of this potential regulatory mechanism should be proven using a real clinical model (chronic or acute inflammatory process, etc.) with an analysis of the endogenous NF-κB targets and corresponding biochemical and physiological changes.

It is noteworthy that NF-κB is not the only signal that co-regulates miR-497 and miR-146a. Both miRNAs belong to the clusters that are negatively regulated by Myc [52]. Their proposed tumor-suppressive properties, inferred from ectopic overexpression experiments [52], would be consistent with the role of negative regulators of NF-κB pathway, which is known to contribute to survival of cancer cells. The complex and coordinated control of these miRNAs could be taken as yet additional evidence of their significance for the cell fate and function, a topic worthy of further exploration.

## MATERIALS AND METHODS

### Cell culture

3B4.1 and C6TA4 (including the derivatives that express mutant IκBα) have been described earlier [28]. Cell culture conditions were as described [53]. The cells were free of contamination by mycoplasma (MycoAlert test from Lonza Group Ltd.) and replication-competent retroviruses [54, 55].

For IL-1β treatment, cells were plated at a density of 2 × 10^6^ cells per 6cm plate and allowed to grow for about 16 hours before cytokine treatment. IL-1β was added at a concentration of 2 ng/mL. After IL-1β treatment, cells were trypsinized, washed, pelleted, and stored at −80°C.

### microRNA profiling and sequencing

3B4.1 cells were cultured and treated with +/− doxycycline. MiRNAs were prepared, sequenced and enumerated as described before [40]. miRNA sequencing data was normalized by scaling. Total number of reads for all miRNA transcript variants was tallied in each of the RNA samples and then counts for all transcript variants were multiplied by a factor to match the total number of counts in the RNA sample with the highest number of total counts (7, 742, 361).

### microRNA target prediction and pathway mapping

miRNA target genes were predicted using the software TargetScan (http://www.targetscan.org). A spreadsheet of predicted target genes for their respective miRNA's was compiled using a custom R script. The compilation of predicted target genes was then mapped to independent cellular pathways using the KEGG Pathway Analysis tool (http://www.genome.jp/kegg/pathway.html).

### Quantitative PCR

RNA was isolated using *mirVana miRNA Isolation Kit* (Life Technologies, Grand Island, NY) following manufacturer protocol for Total RNA isolation without enrichment for microRNAs.

Primers and kits for reverse-transcription and quantitative-PCR were ordered. TaqMan Small RNA Assay kits were used for miRNAs 146a, 215, 381, 497, and RNU6B (Life Technologies). Quantitative PCR cycle threshold data was converted to change-fold in expression by the “delta delta Ct” method [56] using RNU6B as an internal control.

### Plasmids

pRSV-luc (RSV promoter-luciferase in pGL3-Basic) plasmid was obtained from Addgene (Plasmid #40343).

In order to construct pRSV-luc-IKBKB, a portion of the IKBKB 3′UTR was amplified from genomic DNA of LNCaP cells using the following primers: CGCACTTCTAGACGCCTGTCTGCACACTG and CCT TTCCTACAACCCGATTCCGGCCGGTCGATC. The PCR product was digested with FseI and XbaI and cloned between FseI and XbaI sites of pRSV-luc to create the final plasmid.

In order to construct pRSV-luc-mIKBKB, a two-step PCR strategy was used. First, separate PCR products were produced from pRSV-luc-IKBKB template using two distinct pairs of primers (CTCATAAAGGCCAAGAAGGG and AGGT AAAAACACAATTTTCCGCGCAGTGAAATA, and TCTTTTTATTTCACTGCGCGGAAAATTGTGTTTT and TGTGATCATCTGAACTCATT). Second, the two PCR products were purified, combined and subjected to PCR with a new set of primers (CTCATAAAGGCCAAGAAGGG and TGTGATCAT CTGAACTCATT). The resulting product, along with pRSV-luc-IKBKB, was digested with XbaI and SpeI and used to replace the XbaI/SpeI fragment in pRSV-luc-IKBKB.

### Luciferase reporter assays

The cells were plated in 20 wells of a 96-well plate at a seeding density of 10,000 cells / well in 100 μL of antibiotic-free DMEM. 16 hours after plating, cells were transfected with miR-497 (1 pmol / well) or non-targeting siRNA (1 pmol / well), pRSV-βgal (constitutive β-galactosidase expression vector; 70 ng / well), and an appropriate luciferase reporter plasmid (30 ng / well) using Lipofectamine 2000 (Life Technologies) per manufacturer's protocol. Luciferase activity was measured and normalized for that of β-galactosidase as described before [57].

### IKKβ detection by immunoblotting

500,000 cells/well of 6-well plate was plated overnight. Next day the cells were transfected with 100 nM of siGlo (fluorescently labeled negative RNAi control; GE Dharmacon, Lafayette, CO) or miR-497-5P using Lipofectamine 2000 reagent (Life Technologies). Typically, transfection rates of 80–90% were achieved. 48 hrs later the cells were lysed in RIPA buffer and protein concentrations were measured using DC Protein Assay (Bio-Rad). 30 ug of total proteins were then separated on 4–20% Mini-PROTEAN TGX Gel (Bio-Rad) and transferred to PVDF membrane. The membrane was blocked with 5% milk and hybridized with a rabbit polyclonal anti-IKKβ antibody (sc-7607; Santa Cruz Biotechnology, Dallas, TX), or with a mouse monoclonal anti-β-actin antibody as a loading control (A3854; Sigma-Aldrich, St. Louis, MO). After subsequent probing of the membrane with goat anti-rabbit IgG-HRP (sc-2004; Santa Cruz Biotechnology) and goat anti-mouse IgG-HRP (sc-2005; Santa Cruz Biotechnology), respectively, the proteins were visualized using Western Lighting PLUS-ECL reagents (Perkin Elmer, Waltham, MA).

## SUPPLEMENTARY TABLE


